# Beneficial Soil Bacteria: Many Recipes to Promote Plant Growth and Protection

**DOI:** 10.3390/biology14020169

**Published:** 2025-02-07

**Authors:** Ana Alexandre, Kathrin Wippel

**Affiliations:** 1 MED (Mediterranean Institute for Agriculture, Environment and Development) & CHANGE (Global Change and Sustainability Institute), Department of Biology, School of Science and Technology, Universidade de Évora, Pólo da Mitra, Ap. 94, 7002-554 Évora, Portugal; 2 Max Planck Institute for Plant Breeding Research, Department of Plant-Microbe Interactions, 50829 Cologne, Germany

This Special Issue includes studies originating from various countries around the globe and covers diverse types and mechanisms of microbial beneficial functions for plant life. Overall, these publications contribute to enhance our understanding of how soil microbial activity influences plant growth, particularly under abiotic or biotic stress conditions. This area of research is highly valuable not only from the agricultural point of view, but also from the ecological perspective, in order to better understand natural systems.

In the last decade, it became increasingly clear that plant-associated microbial communities as a whole serve as a crucial genetic extension to the host, providing additional beneficial functions. Most of the plant microbiota members originate from the soil, implying that a balanced soil microbiota is essential for healthy and stable ecosystem functioning. Consequently, even a small consortium of microorganisms can exceed the performance of individual strains. Zhang et al. [1] investigate this added value when searching for solutions to fight the devastating clubroot disease in China, caused by a protist infecting Chinese cabbage and leading to severe yield losses that can only temporarily be contained through the application of harmful chemical pesticides. *Plasmodiophora brassicae* forms galls on the cabbage roots and thus blocks water and nutrient uptake in the plant. In their study, the authors employ one *Bacillus* and two *Lysobacter* strains to show that, upon inoculation, a consortium of the three bacteria exhibits the highest biocontrol effect compared to single strains or intra- and inter-genus co-cultures. This seems to be mediated by lowering soil acidity, and by balancing the indigenous microbiota. Importantly, the small consortium even leads to a smaller disease index than treatment with a commercial fungicide, and the cabbage yield is comparable to that of a resistant plant variety.

In addition to pathogen outbreaks, the changing climate affects ecosystems, which has strong implications for all organisms. Soil microbial activity is a reliable indicator for the robustness and resilience of ecosystems to perturbances. Since forests are important environments that mitigate adverse effects of climate change, Walkiewicz et al. [2] set out to understand how different forest types influence this robustness. They sampled soil from different forests in Poland, including two coniferous, two deciduous, and two mixed stands, and each pair was composed of a younger and an older stand. Measurement of several microbial parameters, such as basal respiration, microbial biomass, soil metabolic quotient, and dehydrogenase activity, revealed that mature deciduous forests showed the highest consistency of high microbial activity across the yearly seasons and may therefore be the most sustainable forest stands.

Another man-made threat to the viability of the environment is the extensive usage of chemical fertilizers and pesticides in agriculture to ensure sufficient crop yield for an ever-growing world population. Seenivasagan and Babalola [3] summarize in their review article which microbial alternatives are available for more sustainable practices, and how they function biologically, and provide a comprehensive listing of individual genera or species. They describe mechanisms of a range of bioinoculants, including nitrogen-fixing and sulfur-dissolving bacteria, and microorganisms solubilizing phosphate, potassium, or zinc, and explore the beneficial microbial activities such as production of plant hormones, siderophores for iron chelation, heavy metal detoxification, and antibiotic synthesis. Furthermore, the carrier material for efficient bioinoculant application is discussed, which is crucial for effective agricultural practice.

Scientific reports on the use of plant growth-promoting bacteria (PGPB) to protect important agricultural crops from biotic stress have been accumulating, with high potential to avoid or decrease the use of synthetic pesticides. Among the pathogens that may severely affect plant growth and crop production, plant viruses are an important class and yet probably the least studied in terms of PGPB beneficial effects. Abdelkhalek et al. [4] describe a protective effect of a strain of *Paenibacillus polymyxa* in protecting squash plants from *Zucchini yellow mosaic virus* (ZYMV). This strain (*P. polymyxa* SZYM) was isolated from the rhizosphere of squash plants (*Cucurbita pepo* L.) and its culture filtrate (SZYM-CF) was tested for protection against ZYMV infection in both preventive and post-virus inoculation stages. Greenhouse experiments showed that SZYM-CF was effective in delaying the symptoms from the viral infection. Moreover, the preventive treatment (24 h before viral inoculation) was particularly beneficial considering both plant growth and physiological parameters. In addition, the expression of several plant genes associated with plant protection and defense mechanisms was evaluated and the results corroborated the efficacy of the preventive treatment. This study also analysed the chemical composition of the *P. polymyxa* SZYM culture filtrate and several biologically active aromatic compounds were identified, which enable further experiments that are needed to depict which compound(s) is responsible for the anti-viral protection.

Drought stress is an important global problem that severely impacts agriculture. Water availability directly affects agricultural production and consequently food security. According to FAO reports, 80% of drought-related losses fall on the agricultural sector [5]. Climate change increased the frequency of extreme climate events, including drought episodes. Abdelaal et al. [6] prepared an up-to-date review on the importance of PGPB in alleviating drought stress on plants. This review gives relevance to a better understanding of the plant response to water scarcity, exploring the morphological and anatomical aspects, as well as physiological and biochemical mechanisms of this response. This first part of the review sets the appropriate background to a deeper understanding of the potential use of PGPB in plant drought stress alleviation. It is noteworthy that PGPB may benefit plant tolerance to stress through direct bacterial activity (for example, the production of exopolysaccharides), and also by interfering with the plant drought tolerance mechanisms, such as inducing the levels of phytohormones or defense-related proteins. In this context of improving plant drought stress tolerance, Ahmed et al. [7] isolated bacteria from an agricultural soil in India, with the aim of finding a bacterial strain that could benefit the growth of mung bean (*Vigna radiata* L.) under water deficit conditions. Considering several plant growth-promoting traits and its high tolerance to water stress, isolate PAB19 was further characterized and used in plant inoculation tests under drought conditions. A comprehensive analysis of the inoculation effects of this isolate under different levels of water deficit conditions was performed. Plant growth parameters together with leaf pigment and nutrient analysis supported the positive impact of *Enterobacter* sp./*Leclercia adecarboxylata* PAB19 inoculation (both on control and drought conditions). Since mung beans establish nitrogen-fixing symbiosis with rhizobia, some metrics of this symbiosis were also analysed, and an increase in the symbiotic features was observed. In addition, several plant stress parameters and physiological aspects were evaluated in order to depict changes in the plant response to stress upon PAB19 inoculation.

In a different perspective of PGPB research, Chinachanta et al. [8] investigated the potential of bacteria to enhance commercially valued characteristic of a given crop (not strictly focusing on yield). The economic value of Thai jasmine rice (*Oryza sativa* L.) is highly dependent on its characteristic aroma, which is mainly due the presence of 2-acetyl-1-pyrroline (2AP). Since salinity is a major factor compromising 2AP production, the aim of this study was to isolate soil bacteria able to produce 2AP and with plant growth-promoting traits that would benefit rice plants under salt stress. The *Sinomonas* sp. strain ORF15-23 showed high levels of 2AP production and high tolerance to salinity conditions. Analysis of rice leaves confirmed that inoculation with 2AP-producing salt-tolerant bacteria can increase the plant 2AP content on both control and salinity conditions.

These studies [1,2,3,4,7,8] offer further insights into beneficial microbial functions. Similarly to many publications on the use of PGPB to protect important agricultural crops, they do not yet provide data on field trials, inoculum formulation and safety, which would not only validate the results obtained under controlled greenhouse conditions, but also bring these findings closer to knowledge transfer and to extend farmers’ agronomic options. This will be an exciting future endeavour in the PGPB research community.

## References

[1] Zhang J., Ahmed W., Dai Z., Zhou X., He Z., Wei L., Ji G. (2022). Microbial Consortia: An Engineering Tool to Suppress Clubroot of Chinese Cabbage by Changing the Rhizosphere Bacterial Community Composition. Biology.

[2] Walkiewicz A., Bieganowski A., Rafalska A., Khalil M.I., Osborne B. (2021). Contrasting Effects of Forest Type and Stand Age on Soil Microbial Activities: Local-Scale Variability Analysis. Biology.

[3] Seenivasagan R., Babalola O.O. (2021). Utilization of Microbial Consortia as Biofertilizers and Biopesticides for the Production of Feasible Agricultural Product. Biology.

[4] Abdelkhalek A., Al-Askar A.A., Elbeaino T., Moawad H., El-Gendi H. (2022). Protective and Curative Activities of Paenibacillus Polymyxa against Zucchini Yellow Mosaic Virus Infestation in Squash Plants. Biology.

[5] Pek E., Salman M. (2023). Enabling Pathways for Drought Finance in Agriculture.

[6] Abdelaal K., Alkahtani M., Attia K., Hafez Y., Király L., Künstler A. (2021). The Role of Plant Growth-Promoting Bacteria in Alleviating the Adverse Effects of Drought on Plants. Biology.

[7] Ahmed B., Shahid M., Syed A., Rajput V.D., Elgorban A.M., Minkina T., Bahkali A.H., Lee J. (2021). Drought Tolerant *Enterobacter* sp./*Leclercia adecarboxylata* Secretes Indole-3-Acetic Acid and Other Biomolecules and Enhances the Biological Attributes of *Vigna radiata* (L.) r. Wilczek in Water Deficit Conditions. Biology.

[8] Chinachanta K., Shutsrirung A., Herrmann L., Lesueur D., Pathom-aree W. (2021). Enhancement of the Aroma Compound 2-Acetyl-1-Pyrroline in Thai Jasmine Rice (*Oryza sativa*) by Rhizobacteria under Salt Stress. Biology.

